# Increasing the Efficacy of SLNB in Cases of Malignant Melanoma Located in Close Proximity to the Lymphatic Basin

**DOI:** 10.1155/2014/920349

**Published:** 2014-02-10

**Authors:** Alexander Bogdanov-Berezovsky, Vasileios A. Pagkalos, Eldad Silberstein, Yaron Shoham, Arsinoi A. Xanthinaki, Yuval Krieger

**Affiliations:** Division of Plastic and Reconstructive Surgery, Soroka University Medical Center, Ben-Gurion University, P.O. Box 151, 85101 Beer Sheva, Israel

## Abstract

*Background.* Being predictive of the entire nodal bed, sentinel lymph node biopsy (SLNB) is invaluable in the surgical management of melanoma. Although the concept is simple, sentinel lymph node (SLN) identification and removal can be technically challenging. *Methods.* A total of 102 consecutive patients have undergone SLNB in the Division of Plastic and Reconstructive Surgery of Soroka University Medical Center from 2009 to 2012. Patients have undergone SLNB using a radioactive tracer and blue stain in order to identify the SLN. Although SLNB usually precedes the wide excision of melanoma, primary lesions in close proximity (<10 cm) to the lymph basin require wide excision before beginning the SLN quest. *Results.* All pathology reports confirmed the excision of lymph nodes. *Conclusions.* When treating MM in close proximity to the lymph basin, changing the sequence of the SLNB procedure seems to increase the efficacy of the method.

## 1. Introduction

Malignant melanoma (MM) accounts for only 4% of all malignant neoplasms, but it is responsible for more than 77% of skin cancer deaths [1]. However, despite the fact that the MM incidence has been steadily rising, there has been a decrease in melanoma death rates for patients younger than 65 years in the United States, a finding that likely reflects early detection and improved treatment [2]. According to the American Joint Committee on Cancer (AJCC) (2009), metastasis to regional lymph nodes is the most important prognostic factor in patients with early-stage MM [3].

Sentinel lymph node biopsy (SLNB) for patients with primary cutaneous melanomas was first introduced in the early 1990s [4]. Since then, SLNB has been established as a reliable indicator of the presence of micrometastases in the nodal basin and an accurate prognostic factor in primary melanoma [5]. The concept of the SLNB is based on the principle that all lymphatic fluid from specific tissues is drained to lymph nodes, and as such the first (sentinel) lymph node filtering a specific site can be removed and evaluated for metastasis of malignant cells. Accurate identification of patients with node-negative (stage I or II) or node-positive (stage III) disease improves staging and may facilitate more accurate regional disease control and decision making of treatment with adjuvant therapy and entry to clinical trials [6]. For that reason, surgeons are trying to improve SLNB techniques in order to decrease false-positive rates and permit more specific identification of SLNs [7].

The aim of this study is to reveal certain technical aspects of SLNB emphasizing the management of cases where primary MM is in close proximity to the lymph basin.

## 2. Material and Methods

A total of 102 consecutive patients have undergone SLNB in the Division of Plastic and Reconstructive Surgery of Soroka University Medical Center from 2009 to 2012. In 8 of those patients, the MM location was close to the lymphatic basin and correspondingly to the SLN.

Approximately 12 hours before surgery, the patient is admitted to the nuclear medicine department for the injection of the radioactive tracer. Using a 27 gauge needle, 0.5 mCi of ^99m^Tc sulfur colloid is intradermally injected around the MM site and lymphoscintigrams are obtained between 10 and 150 minutes after the initial injection. The location of the first draining lymph node is detected using a gamma probe and marked on the underlying skin with a permanent marker. In the operating room the surgeon rechecks the SLN location with a gamma probe for proper marking of the planning incision.

With the exception of the cases of face and neck MMs, we use patent blue V (bleu patenté V sodique 2.5% w/v solution; Guerbet) in combination with radioactive tracer. For that reason, approximately 1 cc of dye is intradermally injected to the MM area. The blue dye almost immediately spreads through the surrounding lymphatics and later the SLNB can be visually identified by acquired bluish color.

**Figure 1:**
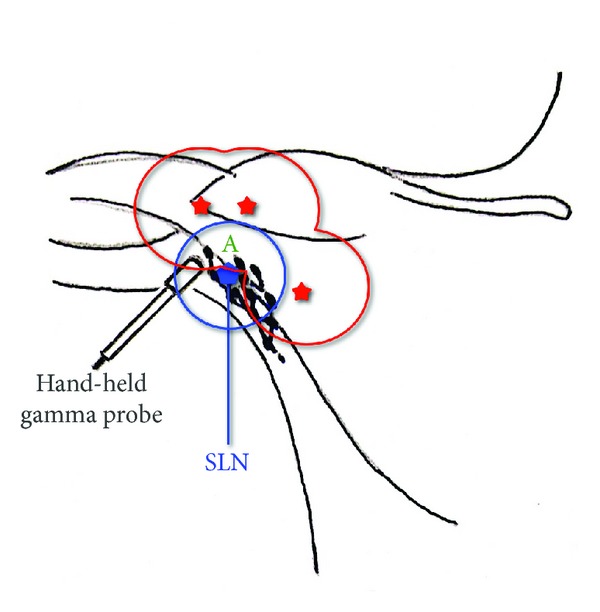
Axillary lymphatic basin: gamma count readings from primary MMs are found to interfere with the readings from the lymphatic basin, thus making tracing of the SLN difficult. SLN: sentinel lymph node; blue circle: range of radiation emitted from the SLN; red stars: possible sites of primary MMs in very close proximity to the lymph basin; red circles: range of radiation emitted from the potential MM sites. Lymph node is marked with “A”; as we can see, this lymph node is inside the range of radiation of both the SLN and the primary melanoma. Since many lymph nodes can be found in similar position, identification of the SLN could be impaired.

An incision is made to the node basin and the quest of blue-stained nodes begins under visual identification and radioactive count is taken with a hand-held gamma probe. We obtain gamma count measurements of the hot spot/node *in vivo* prior to dissection and of the hot spot/node *ex vivo,* after being placed away from the patient's body. Before closing the incision, the tissues immediately adjacent to the removed node are meticulously observed for gamma count readings using a variety of aiming angles and any additional hot nodes are dissected (Figure 1). Excised tissue and radiolabeled lymph nodes are submitted for pathological evaluation.

**Figure 2:**
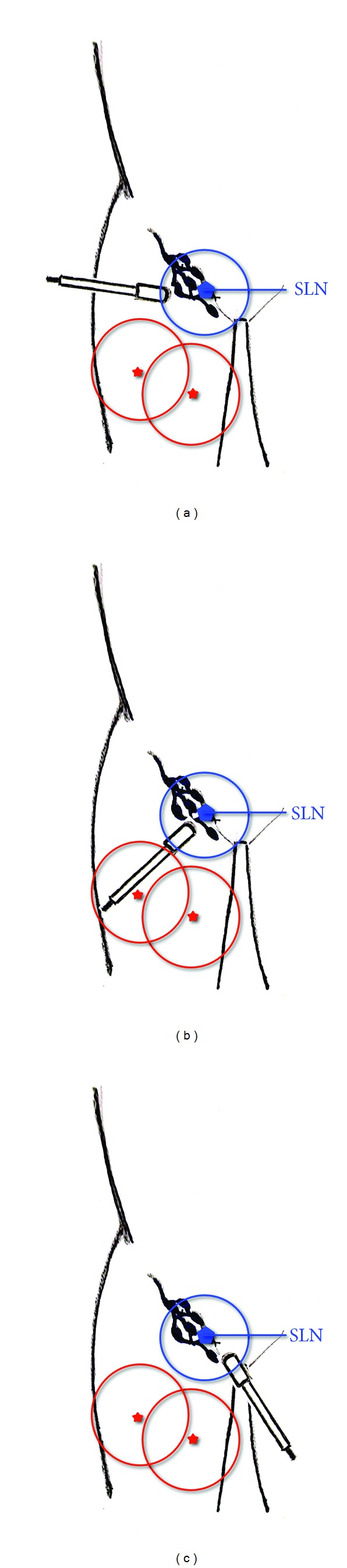
Lymphatic basin of the groin. Example of sites of primary MM in intermediate proximity to the lymphatic basin. Aiming away from the position of the primary MMs can result in clear readings from the hand-held gamma probe. (a), (b) and (c) represent different aiming angles of the probe. SLN: sentinel lymph node; blue circle: range of radiation emitted from the SLN; red stars: possible sites of primary MMs in intermediate proximity to the lymph basin; red circles: range of radiation emitted from the potential MM sites.

Upon successful SLBN, wide excision of MM follows, with excision margins ranging from 1 to 2 cm, depending on Breslow score. Although SLNB usually precedes the wide excision of melanoma, primary lesions in close proximity to the lymph basin require different approach. Gamma count readings from primary MMs with a distance approximately 10 cm or less from the regional lymph nodes are found to interfere with the readings from the lymph basin, thus making tracing of the SLN difficult (Figure 1). In those cases, a previous wide excision of the primary site is found to be highly beneficial for SLN identification. A close proximity with a distance greater than 10 cm,though, will only require to aim the gamma probe in a direction different from the one aiming towards the primary site. Different aiming directions of the gamma probe will allow distinguishing the readings from the nodes and the primary site and therefore provide efficient guidance through the SLNB process without having to excise the MM area first (Figure 2).

## 3. Results

In 8 patients (7.8%), SLN was in close proximity to the melanoma site (maximum distance: 10 cm). All pathology reports confirmed the excision of lymph nodes. The number of lymph nodes excised ranged from 1 to 3. All patients were treated for the MM according to the AJCC guidelines.

## 4. Discussion

Although the concept of SLNB is simple, SLN identification and removal can be technically challenging. For that reason, the use of blue dye alone or in combination with a radiocolloid is invaluable in the intraoperative lymphatic mapping and SLN identification. In a multicenter trial, Morton et al. showed that a standardized SLNB procedure leads to high rates of successful SLN identification, ranging from 95.2% to 99.1% for blue dye alone and blue dye plus radiocolloid, respectively [8]. The SLNB requires a multidisciplinary approach (general surgery, plastic surgery, nuclear medicine, and pathology) and involves multiple parameters such as the agent used, the dose of radiocolloid administered, the time interval between the injection and the surgery, the location of the MM, and the distance of the SLN from the MM.

The combination of intraoperative gamma-probe detection and blue dye raises the sensitivity and specificity of SLNB and has nowadays become the standard of care [9]. However, we have previously reported a case of long-term blue discoloration of patent blue dye as a side effect of SLNB under blue dye guidance [10]. Since skin discoloration can be very frustrating for the patient, especially when sites like the face and neck are involved, we have abandoned the use of patent blue in the face and neck and managed to perform SLNB with the use of radioactive tracer alone.

Regarding the radioactive tracer, technetium-99m (^99m^Tc)-labeled albumin colloid, ^99m^Tc sulfur colloid, or ^99m^Tc human serum albumin are used in the United States, colloidal antimony sulfide is used in Australia, and human albumin nanocolloid is commonly used in Europe [8]. The dose injected at the primary site ranges from 18.5 to 30 MBq (0.5 to 0.8 mCi) and the SLN can be detected 1–30 minutes after injection, depending on the agent and the distance of primary to regional lymph nodes [11]. Previous authors have reported that the radioactive tracer should be injected at a time close to the surgery. They have concluded that the maximum time elapsed between the injection of the radiocolloid and the SLNB is 4 hours and a longer time interval would result in migration of the radiocolloid to nodes beyond the SLN, thus making the differentiation of the SLN unachievable [8, 12]. In our clinical experience, however, injecting the biopsy scar with ^99m^Tc sulfur colloid the day before the surgery did not compromise the SLNB outcomes. We found that identification of the SLN is feasible and the reliability of this practice is supported by the fact that the maximum number of only 3 lymph nodes is identified and dissected from each patient. In cases where the wide biopsy precedes the SLNB, the lymphatic drainage is distracted and this could subsequently minimizes the radioactive counts of the SLN. Leaving maximum time elapsed from the injection of the radioactive tracer to the surgery could serve as a precautious measure in order to obtain sufficient radioactive load in the SLN.

The distance of the MM from the regional lymph basin is a factor that seems to affect the clarity of the gamma-probe readings [7]. Lesion areas injected with radioactive tracer emit radiation at a range that can include or be nearby the SLN (Figures 1 and 2). Although previous authors suggest that in order to keep the original lymphatic drainage pathways undisturbed, SLNB should always precede the wide excision of melanoma [4, 11], our clinical observations suggest a different approach; in cases where the distance of the primary MM from its nodal basin is 10 cm or less, a prevenient wide excision removes the radiation emitting tissue, thus allowing the intraoperative gamma probe to clearly detect radiation readings from the nodal basin only. On the other hand, in cases where MM is located at a distance from the regional lymph nodes that is greater than 10 cm, but still close enough for the gamma readings of the two areas to interfere, a meticulous aiming of the hand-held gamma probe in directions away from the biopsy site will be enough to provide the surgeon with accurate readings.

## 5. Conclusions

Sentinel lymph node biopsy is a highly accurate and low-morbidity procedure that has been endorsed by the AJCC as a valuable staging tool for MM patients who are at risk of clinically occult nodal metastases. Although most of the technical aspects of SLNB have been thoroughly studied, clinical observations have led us to add further refinements to the procedure. Avoiding the use of blue dye in the head and neck region, enlarging the time elapsed between the intradermal radioactive tracer injection and the surgery and, most importantly, introducing the distance of the lymph basin from the biopsy site as a factor defining the excision sequel can optimize our SLNB results.
